# Stem Cell Fate and Immunomodulation Promote Bone Regeneration *via* Composite Bio-Oss^®^/Avitene^TM^ Biomaterial

**DOI:** 10.3389/fbioe.2022.873814

**Published:** 2022-06-27

**Authors:** Maria Rosa Iaquinta, Fernanda Martini, Antonio D’Agostino, Lorenzo Trevisiol, Massimo Bersani, Elena Torreggiani, Mauro Tognon, John Charles Rotondo, Elisa Mazzoni

**Affiliations:** ^1^ Department of Medical Sciences, University of Ferrara, Ferrara, Italy; ^2^ Department of Surgery, University of Verona, Verona, Italy; ^3^ Technological Laboratory for Advanced Therapy (LTTA), University of Ferrara, Ferrara, Italy; ^4^ Center for Studies on Gender Medicine, Department of Medical Sciences, University of Ferrara, Ferrara, Italy; ^5^ Department of Chemistry, Pharmaceutical and Agricultural Sciences, University of Ferrara, Ferrara, Italy

**Keywords:** osteogenic differentiation, immunomodulation, cytokine, chemokine, biomaterial, stem cells

## Abstract

Bone defects in maxillofacial regions lead to noticeable deformity and dysfunctions. Therefore, the use of biomaterials/scaffolds for maxillofacial bone regrowth has been attracting great interest from many surgical specialties and experts. Many approaches have been devised in order to create an optimal bone scaffold capable of achieving desirable degrees of bone integration and osteogenesis. Osteogenesis represents a complex physiological process involving multiple cooperating systems. A tight relationship between the immune and skeletal systems has lately been established using the concept of “osteoimmunology,” since various molecules, particularly those regulating immunological and inflammatory processes, are shared. Inflammatory mediators are now being implicated in bone remodeling, according to new scientific data. In this study, a profiler PCR array was employed to evaluate the expression of cytokines and chemokines in human adipose derived-mesenchymal stem cells (hASCs) cultured on porous hydroxylapatite (HA)/Collagen derived Bio-Oss^®^/Avitene scaffolds, up to day 21. In hASCs grown on the Bio-Oss^®^/Avitene biomaterial, 12 differentially expressed genes (DEGs) were found to be up-regulated, together with 12 DEG down-regulated. Chemokine CCL2, which affects bone metabolism, tested down-regulated. Interestingly, the Bio-Oss^®^/Avitene induced the down-regulation of pro-inflammatory inter-leukin IL-6. In conclusion, our investigation carried out on the Bio-Oss^®^/Avitene scaffold indicates that it could be successfully employed in maxillofacial surgery. Indeed, this composite material has the advantage of being customized on the basis of the individual patients favoring a novel personalized medicine approach.

## Introduction

Bone remodeling is defined by the spatial/temporal coupling of bone resorption and creation. In addition, this biological process is required for skeletal development and appropriate bone structure maintenance. Specifically, bone remodeling is a complex process, which includes bone resorption by osteoclasts and bone formation by osteoblasts, as well as osteocytes which act as mechanosensors/orchestrators of the bone remodeling process (Iaquinta M. et al., 2019).

Approximately 10% of bone fractures do not heal properly (Einhorn and Gerstenfeld, 2015) since the bone regeneration process could fail in extensive bone resections and atrophic non-union (Gao et al., 2014; Ferracini et al., 2018). For this reason, a more efficient clinical therapeutic strategy is needed. In bone tissue engineering (BTE), new biocompatible, osteoconductive and osteoinductive biomaterials/scaffolds, together with stem cells and other factors (Iaquinta M. R. et al., 2019; Iaquinta et al., 2021a; Fakhri et al., 2020; Mazziotta et al., 2021; Mazzoni et al., 2021; Lynch and Lavelle, 2022), are being developed to improve bone repair (Iaquinta M. et al., 2019).

Of the various materials available for scaffolds, titanium alloy, for example, has been employed in dentistry and orthopedic surgery for many years because of its safety and good mechanical properties (Yin et al., 2018). However, titanium is bio-inert. Consequently, it cannot stimulate bone regeneration (Nebe et al., 2008). Calcium phosphates (CaPs), in particular hydroxylapatite (HA), are currently considered gold-standard materials because their composition mimics the mineral bone phase (Spennato et al., 2020). Thus, the purpose of scaffold design should be twofold 1) to provide the required signals for cell proliferation, attachment and function in the setting of natural tissue, as well as 2) to control the immune response (Hortensius and Harley, 2016).

The concept of “osteoimmunology” has been used to define the intimate relationship between the immunological and skeletal systems, suggesting that several molecules, which are involved in the preservation of bone homeostasis and the control of inflammatory functions, are shared (e.g. receptors, signaling molecules and transcription factors) (Chen et al., 2016). Specifically, an emerging role for cytokines and chemokines, as inflammatory mediators, has been highlighted (Galliera et al., 2008). It has been reported that inflammatory cytokines have a negative effect on bone. However, a brief and highly regulated secretion of pro-inflammatory molecules, following acute injury, is considered a critical step for tissue regeneration (Marsell and Einhorn, 2011). In the initial pro-inflammatory response, several molecules, including interleukin-1 (IL1), IL6 and IL11, are involved (Marsell and Einhorn, 2011). IL1 is produced by macrophages and induces production of IL6 in osteoblasts, promotes the production of the primary cartilaginous callus, and promotes angiogenesis at the injured site by activating either one of its two receptors, IL1RI or IL1RII (Marsell and Einhorn, 2011). IL6 stimulates angiogenesis, vascular endothelial growth factor (VEGF) production, and the differentiation of bone cells, both osteoblasts and osteoclasts (Yang et al., 2007). Subsequently, during the resolution of the acute inflammation phase, macrophages are polarized from an M1 phenotype to an M2 phenotype by anti-inflammatory cytokines, such as IL4, IL10, and IL13. Human bone marrow mesenchymal stem cells are attracted locally by cytokines, such as stromal cell-derived factor 1 (SDF1), also known as chemokine C-X-C motif chemokine ligand 12 (CXCL12) (Maruyama et al., 2020). Thus, inflammation is an important biological process that should be considered while creating effective biomaterial-based medicine, since prolonged inflammation can result in delayed wound healing or, in some cases, scaffold rejection and additional tissue damage (Hortensius and Harley, 2016).

In our previous studies, a porous hydroxylapatite/collagen (HA/Collagen) composite biomaterial showed excellent proprieties for both bone grafting and bone regeneration (D’Agostino et al., 2016; Mazzoni et al., 2017; Mazzoni et al., 2020). Specifically, the osteoinductivity properties of the HA/Collagen hybrid scaffold, named Coll/Pro Osteon 200, were investigated in an *in vitro* model of human adipose mesenchymal stem cells (hASCs), whereas the clinical evaluation was carried out in maxillofacial patients (Mazzoni et al., 2019). Since coral reefs are exposed to catastrophic situations, there is a need to look for alternatives (Yahia et al., 2021). Indeed, according to research by the International Union for Conservation of Nature (IUCN), one third of the world’s coral species are said to be at increased risk of extinction. In this context, bovine bone, which is a bio-waste, could be considered a good alternative source of HA for hard tissue replacement in medical and dental therapy (Odusote et al., 2019).

Bio-Oss^®^ is a common bone substitute employed for bone regeneration (Gong et al., 2018). It consists of bovine spongy bone free of organic ingredients, in which the trabecular structure of the fine bone and the internal voids are preserved. Bio-Oss^®^ plays a decisive role in controlling bone regeneration (Gong et al., 2018; Lee et al., 2019; Kosinski et al., 2020). Our previous studies showed that hASCs are an excellent *in vitro* cellular model to assay the proprieties of scaffolds (Mazzoni et al., 2017, Mazzoni et al., 2019; Iaquinta et al., 2021b). For this reason, in our work hASCs were grown on a Bio-Oss^®^/Avitene microfibrillar Collagen scaffold in order to verify how the bone biomaterial can modulate osteoinductivity and immune response.

Existing clinical alternatives do not satisfy all of the criteria for optimum bone scaffolding, thus new materials are being investigated (Jiang et al., 2018). In many cases, it is difficult to form bioceramics into the desired shapes (Raucci et al., 2016). The obtained mixture composed of HA/Collagen, Bio-Oss^®^/Avitene seems very malleable. This characteristic is an important aspect in clinical practice because the prosthesis can be shaped in view of the desired result depending on the patient’s features.

## Materials and Methods

### Bio-Oss^®^/Avitene Scaffold

The Bio-Oss^®^/Avitene scaffold used herein is composed of 1–2 mm bovine spongious bone substitute Bio-Oss^®^ granules (Geistlich Pharma AG) mixed with Avitene Microfibrillar Collagen Hemostat (Bard Warwick, Rhode Island) (Mazzoni et al., 2019). The Bio-Oss^®^ granules (3 g) were combined with 1 g of collagen Avitene, followed by 6 ml of sterile water. The mixture acquired by the combination of Bio-Oss^®^ granules and collagen Avitene was used to obtain small disks (Ø, 1 cm; height, 0.2 cm). These biomaterial blocks were let to dry overnight under UV light.

### Cell Cultures

At the first passage, the human adipose stem cells (hASCs) were bought as cryopreserved frozen cells from Lonza, Milan, Italy (PT-5006). HASCs have surface markers positive for CD13, CD29, CD44, CD73, CD90, CD105, CD166, while, as expected, being negative for other markers, such as CD14, CD31, and CD45. The cells were grown in α-MEM (Lonza, Milan, Italy) supplemented with 10% fetal bovine serum (FBS) (Lonza, Milan, Italy), antibiotics, and incubated at 37°C in a humidified environment with 5% CO_2_. Primary hASC cultures were maintained 1) on the Bio-Oss^®^/Avitene biomaterial and 2) in tissue culture polystyrene (TCPS) vessels (24-well plates, Ø 10 mm), as control (Mazzoni et al., 2019), at a density of 5,000 cells/well. Osteogenic conditions (OC) were obtained utilizing differentiation Bullekit osteogenic medium (Lonza, Milan, Italy), which included osteogenic basal medium (Lonza, Milan, Italy) and osteogenic SigleQuotes, which included dexamethasone, ascorbate, mesenchymal cell growth supplement, l-glutamine, and -glycerophosphate (Lonza, Milan, Italy) (Iaquinta et al., 2021b). The scaffolds were separately arranged in 24-well plates (Ø, 10 mm) to cover the surface area. The scaffolds were then filled with 200 µL of cell suspension containing 10^4^ hASCs for each sample and incubated for 2 h. After this time, a volume of basal medium up to 1 ml was added. The cells were re-fed with fresh medium every three days until the time of analysis.

### Cell Proliferation

The AlamarBlue assay (ThermoFisher Scientific, Milan, Italy) was used to assess the proliferation rate of hASCs grown on the scaffolds (Iaquinta et al., 2021b). As previously reported, the experiment was done to determine the metabolic activity of hASCs when attached (day 0) and cultured on Bio-Oss^®^/Avitene biomaterial and TCPS at day 3, 6, and 9 (Iaquinta et al., 2021b).

The numbers of cells were assessed using a calibration curve consisting of scalar concentration of hASCs (5 × 10^3^-1.6 × 10^4^). Cells were cultured for 3 h at 37°C with a 5% AlamarBlue solution in culture medium. The optical density of the supernatants was then measured using a spectrophotometer at 570 and 620 nm (Thermo Electron Corporation, model Multiskan EX, Helsinki, Finland).

### Cytoskeleton Architecture Evaluation

At day 6, cytoskeletal actin filaments of hASCs grown on Bio-Oss^®^/Avitene were stained with tetramethyl-rhodamineisothiocyanate (TRITC) conjugated-Phalloidin (Sigma, Milan, Italy), as previously described (Mazzoni et al., 2019). Cells were washed with PBS 1X and fixed for 10 min at room temperature (RT) using 10% formalin (Mazzoni et al., 2019). DAPI (0.5 mg/ml) was used to label the nuclei of the cells. The images were captured with a TE 2000-E fluorescence microscope. Digital photos were captured with the DXM1200F digital camera’s ACT-1 and ACT-2 software (Nikon Instruments, Sesto Fiorentino, Italy).

### Cytokine/Chemokine and Osteogenic Gene Expression Profile

A quantitative Real-Time PCR (qPCR) array was carried out in hASCs grown on Bio-Oss^®^/Avitene composite material to identify the genes involved in the immune response while being activated by the scaffold. To this purpose, cells were detached with trypsin–ethylenediaminetetraacetic acid (trypsin-EDTA; Cat. No. BE17-161E; Lonza) from the scaffold Bio-Oss^®^/Avitene and TCPS in order to perform RNA isolation. RNA was isolated using the RNeasy Plus Micro Kit (Qiagen, Milan, Italy), according to the manufacturer’s instructions, at day 21. Total extracted RNA was quantified by using a Nanodrop spectrophotometer (ND-1000; Wilmington, Delaware). Real-time PCR primer sets were utilized to examine the expression of genes encoding 1) human cytokines and chemokines and 2) human osteogenic markers.

The RT^2^ Profiler™ PCR Array Human Cytokines and Chemokines (GeneGlobe ID-PAHS-150Z, Qiagen, Milan Italy) was employed to examine the expression of 84 genes coding for chemokines, interleukins, interferons growth factors, TNF receptor superfamily members and anti-inflammatory cytokines. The RT^2^ Profiler™ PCR Array Human Osteogenesis (GeneGlobe ID—PAHS-026Z, Qiagen, Milan Italy) was used to examine the expression of 84 genes involved in various pathways, such as osteogenic differentiation, cartilage condensation, ossification, bone metabolism, bone mineralization, binding to Ca^2+^ and its homeostasis, extracellular matrix (ECM) protease inhibitors, adhesion molecules, cell-to-cell adhesion, ECM adhesion molecules, and growth factors, as described (Mazzoni et al., 2020). All reactions were performed in triplicate.

### Alizarin Red Staining

Alizarin Red S (AR) staining was used to analyze the calcium deposition by hASCs on scaffolds cultured for 21 days (Mazzoni et al., 2017; Mazzoni et al., 2019). Indeed, matrix mineralization was evaluated by AR staining, whereas its quantification was carried out spectrophotometrically. Briefly, the cells were fixed in 4 wt% paraformaldehyde in PBS 1X for 20 min at room temperature and then washed three times with PBS 1x. The cells were then incubated in 2% (wt/vol) Alizarin Red S solution (Sigma, Milan, Italy) for 20 min at RT. The mineralized substrates were then measured using a water solution containing 20% methanol and 10% acetic acid into cuvettes whereas the matrix mineralization dissolved was read spectrophotometrically (Sigma-Aldrich, Milan, Italy). For each biological sample examined, matrix mineralization was quantified in triplicate. Images were taken using a standard light microscope as described (Mazzoni et al., 2017; Mazzoni et al., 2019; Iaquinta et al., 2021b).

### Statistical Analysis

The *in vitro* experiments were performed in triplicate. Statistical analyses were carried out using Prism6 software (GraphPad 6.0, San Diego, CA, United States). Data obtained from matrix mineralization were analyzed using one-way analysis of variance (ANOVA) with Tukey’s post-test analysis (Mazzoni et al., 2019), while data obtained from the AlamarBlue assay were analyzed with the two-ANOVA, Tukey’s multiple comparisons test. ΔCT value was calculated and *t*-test was used to analyze the Real Time data. The 2^−ΔΔCt^ approach data was used to compute the Fold Change (FC) for each gene expression, while housekeeping genes were employed as controls to normalize data and Log_2_ FC < −1 or >+1 was considered significant. A value of *p*-value < 0.05 was considered significant.

## Results

### Cytocompatibility Analysis of Bio-Oss^®^/Avitene Scaffold Employing hASCs

**FIGURE 1:**
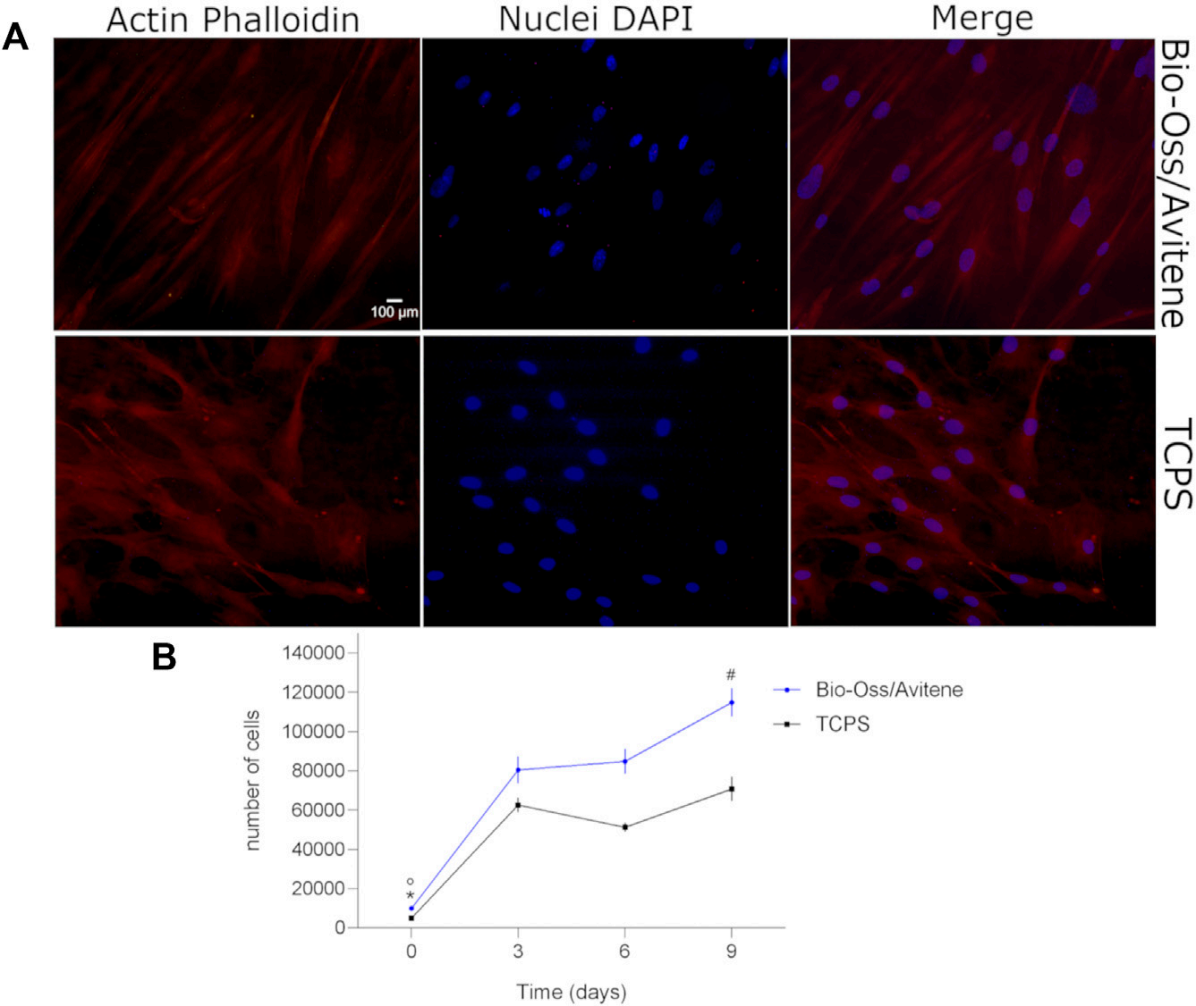
Cytoskeleton architecture and stem cell proliferation. **(A)** Stem cell cytoskeleton architecture. Cell nuclei were stained with 0.5 mg/ml DAPI. Cytoskeleton analysis by Phalloidin TRITC staining was carried out in hASCs grown on the Bio-Oss^®^/Avitene biomaterial (magnification 20 × ). Actin filaments show no alteration in structural organization, confirming the compatibility of the assayed biomaterial, at day 6. **(B)** Human adipose stem cells (hASCs) metabolic activity was evaluated by colorimetric intensity at day 0, 3, 6 and 9 of co-culture on the Bio-Oss^®^/Avitene and culture polystyrene (TCPS) vessels. The biomaterial showed an increase of cell metabolic activity at 3, 6 and 9 days compared to day 0 (°*p*<0.01). hASCs grown on the scaffold showed a statistical increase of cell metabolic activity at day 9 compared to days 3 and 6 (^#^ *p* < 0.01). The metabolic activity measured by AlamarBlue^®^ assay demonstrated different cellular growth kinetics, which are statistically significant at day 3, 6 and 9 compared to cell proliferation on the TCPS control group at day 0 (*p < 0.001). Experiments were performed in technical triplicate for each biological sample (*n* = 3).

The cytocompatibility propriety of the material was evaluated by proliferation/cytoskeleton organization assessment of the hASCs cultured on material at up to day 9. The Bio-Oss^®^/Avitene biomaterial was assessed in terms of cell proliferation at day 3, 6, 9. During the analysis, the Alamar blue test revealed enhanced metabolic activity in hASCs grown on the Bio-Oss^®^/Avitene scaffold, demonstrating that the Bio-Oss^®^/Avitene biomaterial elicited no cytotoxic effects (Figure 1B). After 6 days, actin filaments do not show alteration in structural organization, confirming the compatibility of the assayed biomaterial compared to the control group (Figure 1A).

### Cytokine and Chemokine Gene Expression in hASCs

**FIGURE 2:**
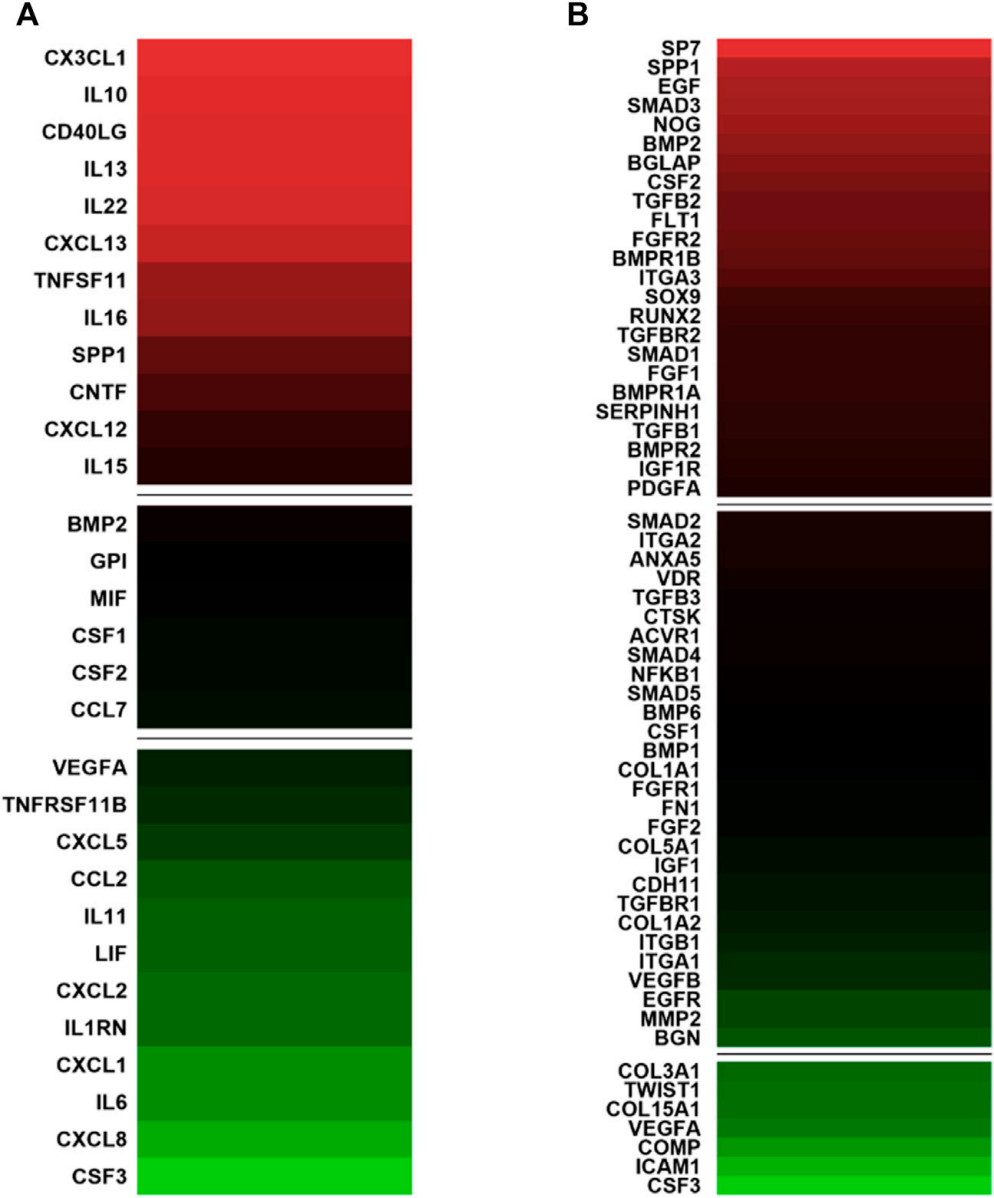
Gene expression involved in immune response and in osteogenic differentiation in human adipose mesenchymal stem cells grown on Bio-Oss^®^/Avitene biomaterial. **(A)** Analysis of genes involved in the immune response compared to tissue cultures in polystyrene (TCPS). In hASC cultures, CX3CL1, IL10, CD40LG, IL13, IL22, CXCL13 TNFSF11, IL16, SPP1, CNTF, CXCL12 and IL15 resulted up-regulated (red). Moreover, VEGFA, TNFRSF11B, CXCL5, CCL2, IL11, LIF, CXCL2, IL1RN CXCL1, IL6, CXCL8 and CSF3 tested down-regulated (green) at day 21. **(B)** PCR array analysis genes involved in osteogenic differentiation. The genes SP7, SPP1, EGF, SMAD3, NOG, BMP2, BGLAP, CSF2, TGFB2, FLT1, FGFR2, BMPR1B, ITGA3, SOX9, RUNX2, TGFBR2, SMAD1, FGF1, BMPR1A, SERPINH1, TGFB1, BMPR2, IGF1R and PDGFA were up-regulated compared to TCPS (red) while COL3A1, TWIST1, COL15A1, VEGFA, COMP, ICAM1, CSF3 resulted as down-regulated after 21 days. A value of *p*-value <0.05 was considered significant. The fold change (FC) of each gene expression was calculated using the 2^−ΔΔCt^ method, whereas housekeeping genes, used as controls, were used to normalize results and Log2 FC; < −1 or > +1 was considered significant). Experiments were performed in technical triplicate for each biological sample (*n* = 3).

The expression profiles of human genes encoding cytokines and chemokines was examined by qPCR Array technology. The hASCs were grown on the Bio-Oss^®^/Avitene scaffold, for 21 days. For data analysis, the Ribosomal protein, large, P0 (RPLP0) was used as a housekeeping gene. Twenty-four DEGs involved in immune response tested either up- or down-regulated. Indeed, in hASCs grown on the Bio-Oss^®^/Avitene biomaterial twelve genes were found to be up-regulated, together with other twelve genes which were down-regulated (Figure 2A; Table 1). The up-regulated genes, which were accounted for included Chemokine (C-X3-C motif) ligand 1 (CX3CL1), Interleukin 10 (IL10), CD40 ligand (CD40LG), Interleukin 13 (IL13), Interleukin 22 (IL22), Chemokine (C-X-C motif) ligand 13 (CXCL13), Tumor necrosis factor (ligand) superfamily, member 11 (TNFSF11), Interleukin 16 (IL16), Secreted phosphoprotein 1 (SPP1), Ciliary neurotrophic factor (CNTF), Chemokine (C-X-C motif) ligand 12 CXCL12 and Interleukin 15 (IL15). The down-regulated genes induced by Bio-Oss^®^/Avitene biomaterial were Vascular endothelial growth factor A (VEGFA), Tumor necrosis factor receptor superfamily, member 11b (TNFRSF11B), Chemokine (C-X-C motif) ligand 5 (CXCL5), Chemokine (C-C motif) ligand 2 (CCL2), Interleukin 11 (IL11), Leukemia inhibitory factor (cholinergic differentiation factor, LIF), Chemokine (C-X-C motif) ligand 2 (CXCL2), Interleukin 1 receptor antagonist (IL1RN), Chemokine (C-X-C motif) ligand 1 (CXCL1), Interleukin 6 (interferon, beta 2 IL6) Chemokine (C-C motif) ligand 8 (CXCL8) and Colony stimulating factor 3 (granulocyte, CSF3).

**TABLE 1:** List of genes involved in immune response found to be up-regulated and down-regulated in hASCs grown on the scaffold at day 21.

Up-Regulated Genes				Down-Regulated Genes			
Number	Symbol/Acronym	Fold-Change(Log_2_ FC)	p-value	Number	Symbol/Acronym	Fold-Change(Log_2_ FC)	p-value
1	CX3CL1	8,56	<0,001	1	VEGFA	-1,15	0,030
2	IL10	8,16	0,001	2	TNFRSF11B	-1,51	0,019
3	CD40LG	8,14	<0,001	3	CXCL5	-2,06	0,003
4	IL13	7,99	0,001	4	CCL2	-2,84	0,010
5	IL22	7,74	0,002	5	IL11	-3,06	0,007
6	CXCL13	7,23	<0,001	6	LIF	-3,18	0,008
7	TNFSF11	5,58	0,002	7	CXCL2	-3,47	<0,001
8	IL16	5,26	0,004	8	IL1RN	-3,47	0,008
9	SPP1	3,57	0,006	9	CXCL1	-4,64	<0,001
10	CNTF	2,73	0,007	10	IL6	-4,64	0,004
11	CXCL12	1,89	0,003	11	CXCL8	-5,64	0,002
12	IL15	1,47	0,039	12	CSF3	-6,64	0,002

Chemokine (C-X3-C motif) ligand 1 (CX3CL1), Interleukin 10 (IL10), CD40 ligand (CD40LG), Interleukin 13 (IL13), Interleukin 22 (IL22), Chemokine (C-X-C motif) ligand 13 (CXCL13), Tumor necrosis factor (ligand) superfamily, member 11 (TNFSF11), Interleukin 16 (IL16), Secreted phosphoprotein 1 (SPP1), Ciliary neurotrophic factor (CNTF), Chemokine (C-X-C motif) ligand 12 (CXCL12), Interleukin 15 (IL15), Vascular endothelial growth factor A (VEGFA), Tumor necrosis factor receptor superfamily, member 11b (TNFRSF11B), Chemokine (C-X-C motif) ligand 5 (CXCL5), Chemokine (C-C motif) ligand 2 (CCL2), Interleukin 11 (IL11), Leukemia inhibitory factor (cholinergic differentiation factor, LIF), Chemokine (C-X-C motif) ligand 2 (CXCL2), Interleukin 1 receptor antagonist (IL1RN), Chemokine (C-X-C motif) ligand 1 (CXCL1), Interleukin 6 (interferon, beta 2 IL6) Chemokine (C-C motif) ligand 8 (CXCL8), Colony stimulating factor 3 (granulocyte, CSF3).

### Bio-Oss^®^/Avitene Scaffold Modulates the Expression of Genes Involved in Skeletal Development in Human Adipose Stem Cells

DEGs (*n* = 31) involved in osteogenic differentiation were detected in hASCs grown on the Bio-Oss^®^/Avitene biomaterial (Figure 2B; Table 2). In hASCs grown on the Bio-Oss^®^/Avitene, DEGs, including 24 up-regulated genes (red) and 7 down-regulated genes (green) were observed. These up-regulated genes included osteoblast differentiation-related genes, for instance, SPP1, SMAD family member 3 (SMAD3), Noggin (NOG), Bone morphogenetic protein 2 (BMP2), the bone morphogenetic protein receptor type II (BMPR2), Bone morphogenetic protein receptor, type IA (BMPR1A), the Bone gamma-carboxyglutamate (gla) protein (BGLAP), while fibroblast growth factor receptor 2 (FGFR2), resulted as up-regulated in hASCs grown on the Bio-Oss^®^/Avitene compared to the control group (TCPS). Up-regulated transcription factors included Runt-related transcription factor 2 (RUNX2), transcription factor Sp7 (SP7) and SMAD family member 1 (SMAD1). Moreover, the transcription factor condensation SRY (sex-related Y)-type high mobility group box SOX-9 (SOX9) and BMPR1B, which plays a central role in chondrocyte differentiation, was also found to be up-regulated in hASCs grown on the scaffold, at day 21. Human cell adhesion analysis and extracellular matrix (ECM) gene expression revealed that the following growth factors were up-regulated in hASCs grown on the scaffold: Epidermal Growth Factor (EGF); colony-stimulating factor 2 (CSF2); (Fibroblast Growth Factor 1 (FGF1); Platelet-derived growth factor subunit A (PDFGA). Furthermore, genes encoding for ECM molecules, adhesion molecules, such as Fms Related Receptor Tyrosine Kinase 1 (FLT1), Integrin alpha-3 (ITGA3) and Serpin Family H Member 1 (SERPINH1) were also up-regulated. The tested genes which resulted as down-regulated included those that code for ECM molecules, such as Col type III alpha 1 (COL3A1), Col type V alpha 1 (COL15A1), Twist Family BHLH Transcription Factor 1 (TWIST), VEGFA, Cartilage Oligomeric Matrix Protein (COMP), Intercellular adhesion molecule 1 (ICAM1) and CSF3.

**TABLE 2:** List of genes involved in osteogenic differentiation found to be up-regulated and down-regulated in hASCs grown on the scaffold at day 21.

Up-Regulated Genes				Down-Regulated Genes			
Number	Symbol/Acronym	Fold-Change(Log_2_ FC)	p-value	Number	Symbol/Acronym	Fold-Change(Log_2_ FC)	p-value
1	SP7	7,14	0,003	1	COL3A1	−1,09	0,032
2	SPP1	5,52	0,004	2	TWIST1	−1,15	0,048
3	EGF	5,12	0,003	3	COL15A1	−1,18	0,027
4	SMAD3	4,99	0,014	4	VEGFA	−1,29	0,042
5	NOG	4,8	0,003	5	COMP	−1,56	0,042
6	BMP2	4,41	0,015	6	ICAM1	−1,84	0,025
7	BGLAP	4,05	0,017	7	CSF3	−2,12	0,023
8	CSF2	3,67	0,007	—	—	—	—
9	TGFB2	3,41	0,082	—	—	—	—
10	FLT1	3,37	0,006	—	—	—	—
11	FGFR2	3,23	0,006	—	—	—	—
12	BMPR1B	2,99	0,031	—	—	—	—
13	ITGA3	2,64	0,009	—	—	—	—
14	SOX9	2,08	0,027	—	—	—	—
15	RUNX2	1,77	0,017	—	—	—	—
16	TGFBR2	1,74	0,082	—	—	—	—
17	SMAD1	1,72	0,033	—	—	—	—
18	FGF1	1,59	0,022	—	—	—	—
19	BMPR1A	1,57	0,091	—	—	—	—
20	SERPINH1	1,52	0,022	—	—	—	—
21	TGFB1	1,42	0,052	—	—	—	—
22	BMPR2	1,3	0,022	—	—	—	—
23	IGF1R	1,23	0,034	—	—	—	—
24	PDGFA	1,1	0,035	—	—	—	—

Sp7 transcription factor (SP7), Secreted phosphoprotein 1 (SPP1), Epidermal growth factor (EGF), SMAD, family member 3 (SMAD3), Noggin (NOG), Bone morphogenetic protein 2 (BMP2), Bone gamma-carboxyglutamate (gla) protein (BGLAP), Colony stimulating factor 2 (CSF2), Transforming growth factor, beta 3 (TGFB2), Fms-related tyrosine kinase 1 (FLT1), Fibroblast growth factor receptor 2 (FGFR2), Bone morphogenetic protein receptor, type IB (BMPR1B), integrin, alpha 3 (ITGA3), SRY (sex determining region Y)-box 9 (SOX9), Runt-related transcription factor 2 (RUNX2), Transforming growth factor, beta receptor II (TGFBR2), SMAD, family member 1 (SMAD1), Fibroblast growth factor 1 (FGF1), Bone morphogenetic protein receptor, type IA (BMPR1A), serpin peptidase inhibitor, clade H (heat shock protein 47), member 1 (SERPINH1), Transforming growth factor, beta 1 (TGFB1), Bone morphogenetic protein receptor, type II (BMPR2), Insulin-like growth factor 1 receptor (IGF1R), Platelet-derived growth factor alpha polypeptide (PDGFA), collagen, type III, alpha 1 (COL3A1), Twist homolog 1 (TWIST1), Collagen, type XV, alpha 1 (COL15A1), Vascular endothelial growth factor A (VEGFA), Cartilage oligomeric matrix protein (COMP), Intercellular adhesion molecule 1 (ICAM1) and Colony stimulating factor 3 (CSF3).

### Biomaterial Induced Matrix Mineralization

**FIGURE 3:**
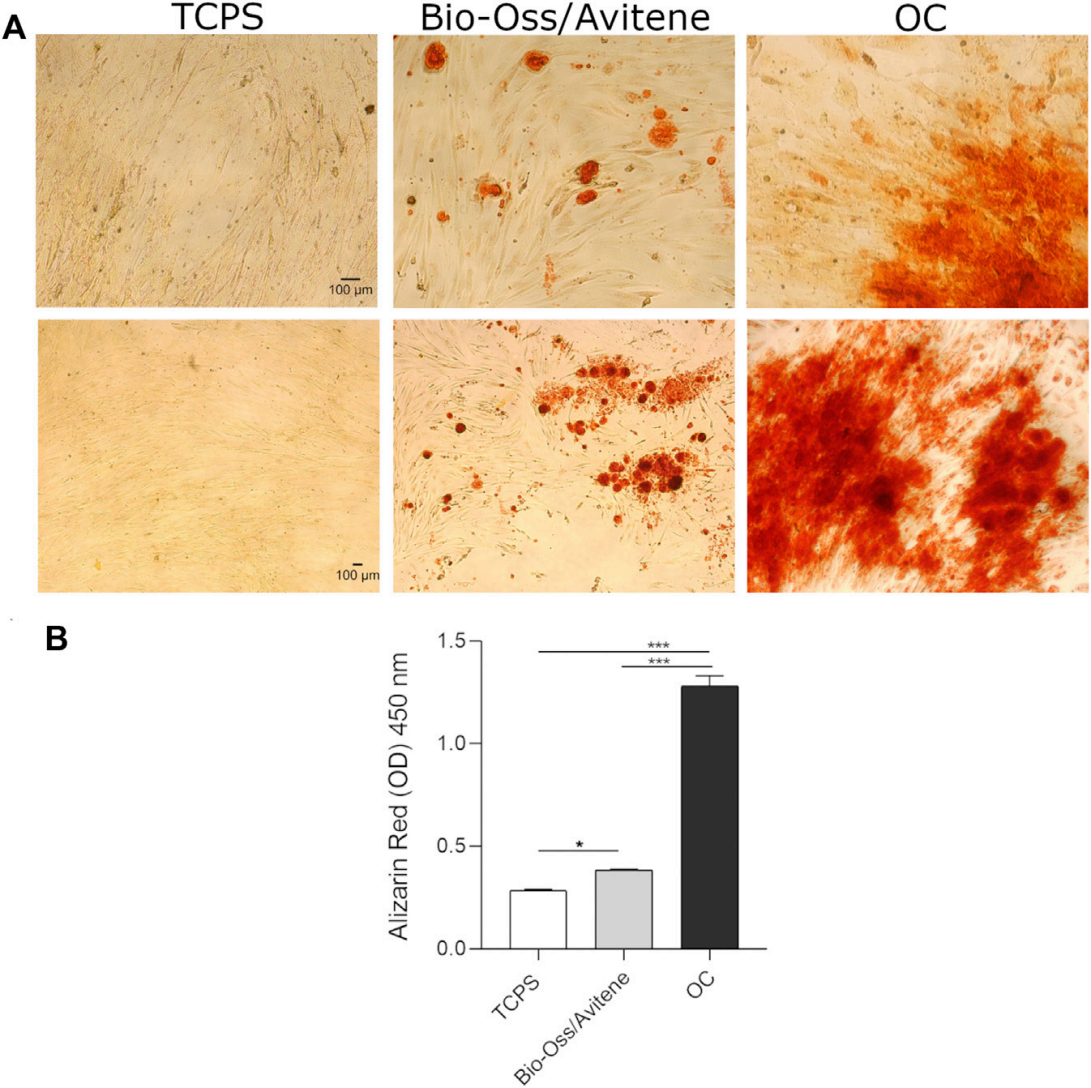
Biomaterial induced matrix mineralization. **(A)** hASCs grown on scaffolds were stained with AR and imaged with bright-field microscopy at day 21 (10× magnification upper figures, 4× magnification lower figures). The biomaterial induces mineral matrix deposition better than the plastic vessel (TCPS), the control. **(B)** The quantification of AR was performed by eluting AR staining and acquiring optical density measurements. Osteogenic differentiation of hASCs grown on the biomaterial was increased compared to TCPS (*p < 0.05). In OC, the calcium deposits were higher than in cells grown on the scaffold and in TCPS (***p < 0.0001). Experiments were performed in technical triplicate for each biological sample (*n* = 3).

The presence of mineralized (calcified) matrix portions was highlighted in hASC cultures using alizarin red staining. Bright-field microscopy was used to examine hASCs growing on scaffolds dyed with AR. The biomaterial stimulates mineral matrix deposition better than the control plastic vessel (TCPS). (Figures 3A,B). The quantification of AR was accomplished by eluting AR stains and measuring its relative optical density. hASCs cultured on the biomaterial showed more osteogenic differentiation than TCPS (∗*p* < 0.05; Figure 3B). It is worth noting that in OC, the deposition of inorganic calcium salts was the most evident, and the calcium deposits in positive control (OC) were higher than in cells grown on the composite material and in TCPS (∗∗∗*p* < 0.0001) (Figure 3B).

## Discussion

In this study, a composite Bio-Oss^®^/Avitene material was assessed utilizing an *in vitro* cellular model comprised of primary hASCs for its biological proprieties. The metabolic activity analysis conducted *in vitro* with hASCs suggests that the Bio-Oss^®^/Avitene scaffold meets the requirements for *in vitro* biocompatibility, offering a good microenvironment for hASCs adhesion and proliferation. Cytoskeleton architecture seemed to be well organized. Indeed, the actin filaments were distributed uniformly in hASCs grown on the Bio-Oss^®^/Avitene and plastic vessels (TCPS). Gene transcript studies were carried out at day 21 by analyzing the main genes involved in the immune response to hASCs grown on the Bio-Oss^®^/Avitene biomaterial, compared to hASCs grown in a plastic vessel, used as control. Many published studies demonstrated the specific effects of certain chemokines on osteoclasts and/or osteoblasts differentiation and function. Bone ECM is constantly remodeled by bone-resorbing osteoclasts and bone-forming osteoblasts. An interaction between bone remodeling and the immune system is supported by several arguments (Brylka and Schinke, 2019) 1) osteoclasts, derived from hematopoietic progenitor cells, represent a highly specialized immune cell, 2) osteoclast and osteoblast progenitors are located in the bone marrow. These cells are in direct contact with progenitor or memory cells of the immune system and 3) the major pro-osteoclastogenic cytokine RANKL is not only expressed by osteoblast lineage cells, but also by activated T cells and B cells, influencing both osteoclast differentiation and other immune cell types (Brylka and Schinke, 2019). The goal of a new scientific field called osteoimmunology, which is important in the context of inflammation-induced bone disorders and defective bone remodeling, is to better understand these influences.

Cytokines are small signaling proteins secreted by immune cells and other cell types to induce immune response, inflammation, and other functions/process. Historically, cytokines were divided into two functional groups: lymphokines/interleukins and chemokines. All cytokines released by immune cells were called lymphokines/interleukins, whereas chemotactic cytokines were called chemokines. Up-regulated genes were found to include anti-inflammatory cytokine expression, such as IL-13 and IL-22 in hASCs grown on the Bio-Oss^®^/Avitene biomaterial. IL-22 is involved in human MSC proliferation/migration in inflammatory environments (El-Zayadi et al., 2016). This biomaterial also induced CD40L up-regulation, which facilitates B-cell activation to promote early bone healing (Duvvuru et al., 2019). SPP1 gene, which codifies for osteopontin (OPN), was up-regulated by the Bio-Oss^®^/Avitene biomaterial. OPN is considered to play an important role in bone regrowth (Mazzoni et al., 2019). According to several pieces of research, OPN acts as a cell adhesive, signaling, migratory, and survival stimulant for a variety of mesenchymal, epithelial and inflammatory cells, as well as a potent regulator of osseous and ectopic calcification. Based on these reports, a general picture of OPN as an important inflammation and biomineralization regulator is emerging (Giachelli and Steitz, 2000). Recently, Mahon et al., observed increased expression of BMP2, ALP and OPN in MSCs in the presence of recombinant anti-inflammatory cytokine IL-10 (Mahon et al., 2020), demonstrating a direct pro-osteogenic role for this cytokine, which resulted as up-regulated by the Bio-Oss^®^/Avitene biomaterial in our study. Current strategies being explored include incorporating anti-inflammatory cytokines, including IL-10, into scaffolds (Holladay et al., 2011). Protein XC chemokine ligand-13 (CXCL13) and its receptors were involved in the process of bone marrow MSC (BMSCs) migration. CXCL13, for example, along with the chemokine CXCR5, influenced B-cell chemotaxis and BMSC recruitment during fracture repair (Tian et al., 2015; Jiang et al., 2018). CNTF resulted as over-expressed in hASCs grown on the Bio-Oss^®^/Avitene biomaterial, after day 21. The CNTF gene belongs to the IL family (interleukin)-6-type cytokines together with IL-6, IL-11 and LIF. These cytokines stimulate the expression of target genes involved in differentiation, survival, apoptosis and proliferation. Members of this family have both pro- and anti-inflammatory qualities, and they play important roles in hematopoiesis, as well as acute-phase and immunological responses in the organism (Heinrich et al., 2003). Neutrophils perform an important initial role in infection controls, first by phagocytosing pathogens and then by releasing mediators that attract more leukocytes into the injured tissue. It is therefore important to understand how these cells are recruited. Chemokine CXCL1 and many others are potent chemo-attractants, which neutrophils respond to (De Filippo et al., 2013). In our study, CXCL1 resulted as down-regulated in hASCs grown on the Bio-Oss^®^/Avitene biomaterial, compared to the control. CXCL8 (also known as Interleukin 8) binds to CXCR1 as well as CXCR2, specifically (Lazennec and Richmond, 2010; Pu et al., 2017). CXCL8 also tested as down-expressed in hASCs grown on the Bio-Oss^®^/Avitene biomaterial, after 21 days. CXCL8, a multifunctional pro-inflammatory chemokine that was initially classified as a neutrophil chemoattractant, has recently been found to be a key contributor in tumorigenesis (Asokan and Bandapalli, 2021). Indeed, CXCL8 is up-regulated in several human cancers. This shows that the tumor and its microenvironment interact, promoting tumor growth by increasing angiogenesis, tumor genetic diversity, survival, proliferation, immune evasion, metastasis, and multidrug resistance (Asokan and Bandapalli, 2021).

Several chemokines have a considerable favorable impact on osteoclastogenesis. A recent investigation demonstrated that CXCL2 might also inhibit osteoblast differentiation (Yang et al., 2019). In this study, hASC cultures grown on Bio-Oss^®^/Avitene composite material showed down regulated expression of cytokines that promote bone resorption, including CXCL2 (Table 1).

Chemokine CCL2 tested as down-regulated in hASCs grown on the Bio-Oss^®^/Avitene. CCL2 can affect bone metabolism. Osseous inflammation studies have shown selective expression of this chemokine by osteoblasts, which are strictly correlated to monocyte recruitment at osteolytic inflammatory lesion sites (Galliera et al., 2008). *In vivo*, CCL2 is one of the main chemokines induced in osteoblasts in response to bacterial infections (Galliera et al., 2008).

Pro-inflammatory cytokine IL-6 (Duvvuru et al., 2019) expression decreased in hASCs grown on the Bio-Oss^®^/Avitene scaffold under analysis. Gene transcript studies of genes involved in the osteogenetic pathway indicate that DEGs include induced/up-regulated osteoblast markers. The Bio-Oss^®^/Avitene composite biomaterial positively modulated osteoblast differentiation genes, such as SPP1, SMAD3, BMP2 and BGLAP and TGFβ1. SPP1 gene over-expression is also confirmed upon analyzing hASC osteogenic gene expression profile when grown on the Bio-Oss^®^/Avitene, at day 21. BMP and BMPRs are known as important factors for skeletal development, regeneration and homeostasis (Iaquinta et al., 2021a). BMP2 is involved in bone production, bone remodeling, bone development, and osteoblast differentiation, according to research. SPP1 and BMP2 upregulation could correlate to IL-10 over-expression (Mahon et al., 2020). BGLAP gene encodes for osteocalcin (OCN), a very abundant bone protein released by osteoblasts that affects bone remodeling and energy metabolism (Wang et al., 2021). DEGs include the TGFβ1 gene that codifies an ubiquitous growth factor in skeletal tissue, playing a major role in development and maintenance of bone metabolism by controlling cellular proliferation, differentiation, matrix deposition and migration (Janssens et al., 2005). MSC recruitment is a critical step in the formation and maintenance of and repair of tissues throughout the body. In this context, TGFβ1 is a potent chemokine, which is essential for MSC recruitment in bone, as it couples the remodeling cycle. Dysregulation of TGFβ signaling or cilia has been linked to a number of skeletal pathologies (Labour et al., 2016). In our research, the expression of transcription factors Runx2 and SP7 increased at day 21. Runx2 is essential for osteoblast differentiation and chondrocyte maturation. During osteoblast differentiation, Runx2 is weakly expressed in uncommitted MSCs, and its expression is up-regulated in pre-osteoblasts, where it is most abundant in immature osteoblasts and is least abundant in mature osteoblasts (Komori, 2019). Osterix (OSX), also known as SP7, is an osteoblast-specific transcription factor. (Tang et al., 2011). It controls maturation in functional osteoblasts and subsequent differentiation to osteocytes in the latter phases of osteogenesis and maturation. Deletion of Osx in mice leads to neonatal lethality due to a failure in general bone formation, severe rib cage malformation and a lack of expression of osteoblast genes, such as Sparc and SPP1 (Tang et al., 2011). As a result, during osteogenic lineage specification, RUNX2 enhances mesenchymal progenitor differentiation, thus initiating osteogensis while OSX promotes the maturation of functional osteoblasts. We detected the up-regulation of SOX9 transcription factor and BMPR1B gene expression in hASCs grown on HA/Collagen scaffolds. BMPRIB is most abundant in mesenchymal precartilage condensations, developed osteoblasts, and chondrocytes. The knockdown of BMPR1b by siRNA inhibited the osteogenic differentiation of human MSCs (Wang et al., 2017). SOX9 is a transcription factor which plays a key role in chondrogenesis, by controlling type II collagen and aggrecan expression, as well as supporting chondrocyte survival and hypertrophy (Lefebvre and Dvir-Ginzberg, 2017). It is interesting to evaluate the differentially expressed genes modulated from BioOss^®^/Avitene in comparison with other materials with similar chemical-physical characteristics.

Scaffold osteoinductive capability is revealed in this work by matrix mineralization identified in hASCs grown on the scaffold at day 21. The composite material (BioOss^®^/Avitene) has the advantage of being customized as it is created on the basis of the individual patients and this ensure novel personalized medicine.

## Conclusion

In order to develop new biomaterials, an in-depth understanding of a number of relevant issues is mandatory. Specifically, it is significantly important to foresee what effects implanted biomaterials may induce on osteogenesis and the immune environment/system. Our *in vitro* results have enabled us to better understand the effect of the Bio-Oss^®^/Avitene scaffold used in maxillo-facial surgery. Bio-Oss^®^/Avitene reduced the expression of several inflammatory cytokines. Indeed, it displayed no significant effects on inflammation. Further analysis will be needed to define the pathway involved in the immune response. However, the obtained results demonstrated that composite Bio-Oss/Avitene has immunomodulatory potential and is capable of directing anti-inflammatory innate immune-mediated responses that are associated with tissue repair. The Bio-Oss^®^/Avitene biomaterial allowed hASC proliferation and differentiation to be enhanced by inducing the up-regulation of genes involved in osteogenic pathways. In conclusion, the study carried out on the Bio-Oss^®^/Avitene scaffold indicates that it could be a suitable material for use in maxillo-facial surgery.

## Data Availability

The original contributions presented in the study are included in the article/Supplementary Material, further inquiries can be directed to the corresponding author.
